# Qualitative systematic reviews to increase the volume and diversity of patient perspectives included in the development of core outcome sets. Tuberculosis: a pilot study

**DOI:** 10.1186/1745-6215-16-S1-O3

**Published:** 2015-05-29

**Authors:** Lucy Elizabeth Hoppe

**Affiliations:** 1 School of Dentistry (Cochrane Oral Health Group), University of Manchester, Manchester, UK; 2 Centre for Clinical Practice, National Institute for Health and Care Excellence, London, UK

## Background

Patient involvement is a core value of contemporary healthcare, and an emerging component of core outcome set (COS) methodology.

This project pilots the use of qualitative systematic reviews of patient perspectives on outcome prioritisation in COS development, specifically for a COS for tuberculosis. The fight against tuberculosis has been hampered by the burden of treatment regimens and drug resistance, and clinical trials are ongoing. A COS for tuberculosis will ensure that outcome selection across trials is consistent, free from selection bias and relevant to patients, clinicians and policy-makers.

## Methods

MEDLINE, EMBASE and ASSIA were searched for studies exploring patient perspectives on tuberculosis outcomes and their value. Each search strategy included terms for 1) qualitative research and 2) tuberculosis.

Inclusion criteria: participants of any age with a diagnosis of tuberculosis; direct contact or observation; any geographical location; publication after 2003 and in English.

Studies were appraised using the CASP checklist. Methods for synthesis should be based on the aims of review. Here, the aim is not model or theory generation, but an aggregation of perspectives and experiences. Thematic analysis was therefore used.

## Findings

13 papers were identified [1-13].

Searching for studies was demanding due to inadequate qualitative indexing, non-meaningful titles and poor abstracts. Data extraction was also complex, with substantial irrelevant data.

The included studies were conducted in countries across South America, Africa and Asia. This improved the international relevance of the findings, though perspectives from Europe and North America, as well as more socioeconomically developed areas, was lacking. Participants ranged in age from 5 to 80, and included an approximately equal number of men and women. There was limited coverage of individuals who had defaulted or failed treatment.

Many participants used indigenous knowledge in place of biomedical knowledge, leading to difficulties in interpretation.

Outcomes that impair physical and social functioning are emphasised, with their importance often linked to fear or stigma. Mortality and treatment success or failure, as well as the adverse effects of treatment, were noted. Despite this, they are not consistently reported in previous clinical trials. For example, a Cochrane Review [14] into the use of rifabutin for active tuberculosis found that only 2 of 8 RCTs reported cure and 4 of 8 reported adverse events.

No studies were designed with outcome prioritisation in mind. The evidence was largely ‘indirect’ and ‘concealed’ in the results, reflecting the lack of conceptualisation of the phenomenon (patient outcome prioritisation) and novelty of the research area. The paucity of direct information that explicitly explores patient perspectives on the prioritisation of treatment outcomes is justification for future qualitative research.
